# Implementation of a Standardized Nurse‐Guided Heparinization Protocol Improves Anticoagulation During Left Atrial Ablation Procedures

**DOI:** 10.1111/jce.70378

**Published:** 2026-05-18

**Authors:** Vanessa Sciacca, Jasmin Retzlaff, Martin Braun, Thomas Fink, Angeliki Darma, Yuri Bocchini, Karen Harutyunyan, Philipp Lucas, Nadica Trajkovksa, Denise Guckel, Moneeb Khalaph, Maximilian Moersdorf, Maxim Didenko, Christian Sohns, Philipp Sommer

**Affiliations:** ^1^ Department of Electrophysiology, Heart‐ and Diabetescenter NRW Ruhr‐University Bochum Bochum Germany

**Keywords:** ACT, anticoagulation, atrial fibrillation, catheter ablation, heparin, nurse‐led protocol, workflow

## Abstract

**Background and Aims:**

Effective intraprocedural anticoagulation is essential during catheter ablation of atrial fibrillation (AF) and left atrial tachycardia (AT) procedures. This study evaluated a novel, standardized, nurse‐led heparin protocol regarding anticoagulation performance and safety.

**Methods:**

Consecutive patients undergoing AF or AT ablation between May 2022 and 2023 were treated using a standardized heparinization protocol managed by electrophysiology nursing staff (study group) and compared with consecutive patients undergoing ablation between May 2021 and April 2022, in whom heparin was administered at the operator's discretion (control group). Patients in the study cohort received an initial dose of 5000 IU heparin after venous access and a supplemental bolus (1000 IU/10 kg above 50 kg bodyweight) after transseptal puncture. ACT was then assessed every 20 min using a point‐of‐care coagulation device in both groups. Repeat heparin administration was autonomously performed by nurses in the study group following a standardized protocol. The primary endpoint was the proportion of patients in whom at least one intraprocedural ACT measurement exceeded 300 s at any time during the procedure.

**Results:**

Each group included 655 patients with comparable baseline characteristics. The study group more frequently achieved the therapeutic target (ACT > 300 s, 84.6% vs. 59.7%, *p* < 0.0001), reached therapeutic ACT faster, and showed fewer exclusively subtherapeutic ACT values. Excessive anticoagulation was less common (ACT > 400 s: 2.0% in the study group vs. 5.0% in the control group, *p* = 0.0027). Complication rates were low and similar in both groups.

**Conclusions:**

A standardized, nurse‐led heparinization protocol improved the speed, consistency, and precision of intraprocedural anticoagulation during left atrial ablation without increasing procedural complications.

## Graphical abstract

A standardized, nurse‐led heparinization protocol significantly improves the speed, consistency, and precision of achieving therapeutic ACT levels during left atrial ablation, while reducing excessive anticoagulation and maintaining a comparable safety profile to conventional operator‐guided dosing.

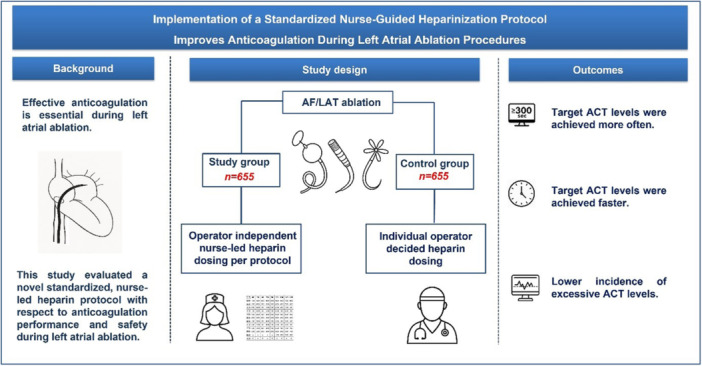

## 1 Introduction

Atrial fibrillation (AF) is the most prevalent sustained cardiac arrhythmia worldwide, affecting more than 33 million individuals [1, 2]. Today, catheter ablation has become a key strategy of rhythm‐control in symptomatic patients with AF [1, 2]. Despite significant technological advancements in left atrial catheter ablation, thromboembolism remains a significant procedural risk, necessitating vigilant anticoagulation management and procedural monitoring [3]. The thromboembolic risk associated with left atrial ablation is multifactorial. Transseptal access facilitates direct communication between the systemic venous system and the left atrium, thereby providing a nidus for thrombus formation. Intraluminal catheter manipulation promotes localized blood flow disruption and stasis, while energy delivery induces endothelial denudation and exposure of subendothelial procoagulant matrix proteins. These convergent mechanisms potentiate in situ thrombus formation and subsequent embolization. Clinically, this may manifest as transient ischemic attack (TIA), ischemic stroke, or subclinical cerebral embolic lesions [3, 4]. To mitigate thromboembolic risk, periprocedural anticoagulation strategies have evolved substantially. International guidelines recommend the use of intravenous unfractionated heparin (UFH) to maintain an intraprocedural activated clotting time (ACT) > 300 s throughout the procedure [1, 2]. Despite these recommendations, real‐world practice exhibits significant heterogeneity [5, 6, 7, 8, 9]. Dosing regimens vary widely between operators, ranging from fixed boluses to weight‐based strategies and varying ACT measurement intervals. Delays in achieving therapeutic ACT early in the procedure are common [4, 6, 7]. These delays represent periods of increased thromboembolic vulnerability [10]. This study evaluates the impact of a novel standardized protocol for nurse‐guided heparin dosing during left atrial ablation procedures in terms of anticoagulation efficacy and safety compared with individual operator‐guided heparinization.

## 2 Methods

### 2.1 Study Design

This was an observational single‐center study designed to compare two distinct strategies for intraprocedural anticoagulation management during left atrial ablation: a newly implemented standardized nurse‐led heparinization protocol (study group) and conventional operator‐guided heparin dosing (control group). The study group consisted of consecutive patients undergoing left atrial ablation for AF or atrial tachycardia (AT) between May 2022 and 2023. Patients who received left atrial ablation between May 2021 and April 2022 without the use of a standardized heparin protocol served as a control group. Both groups were managed by the same electrophysiology teams, using identical procedural techniques and periprocedural care, with intraprocedural heparin dosing strategy as the only variable. The study was conducted at a high‐volume tertiary referral electrophysiology center. As this was an observational study including consecutive patients over predefined time periods before and after implementation of the protocol, no formal a priori sample size calculation was performed. The study population was determined by the total number of eligible patients treated during the respective study periods. The research protocol was approved by the institutional ethics committee (ethical review board file number 2019–563) and conducted in accordance with the Declaration of Helsinki. All patients provided written informed consent for inclusion in the study and for data collection related to procedural parameters and clinical outcomes.

### 2.2 Heparinization Protocol

**Figure 1:**
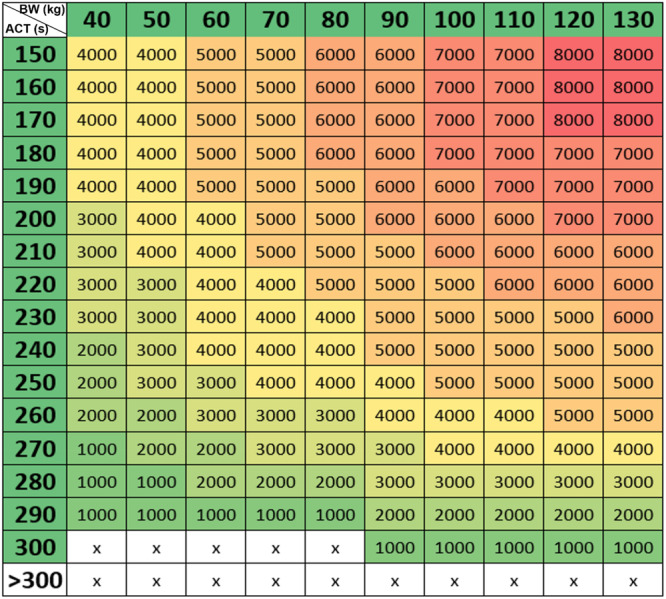
Heparin dose adjustment scheme based on ACT and body weight. Nomogram for heparin dose adjustments based on activated clotting time (ACT) and patient body weight. The table shows the recommended heparin bolus dose (in IU) to achieve therapeutic anticoagulation during the procedure, stratified by ACT (seconds) and patient weight (kg). Green indicates lower doses, yellow indicates intermediate doses, and red indicates higher doses.

The heparinization protocol (Figure 1) was developed with the aim of achieving rapid and sustained effective anticoagulation while streamlining intraprocedural workflows during left atrial ablation procedures. Its primary objectives were to reach an ACT of more than 300 s as quickly as possible following transseptal puncture and to maintain ACT consistently above this threshold throughout the procedure. Furthermore, the heparinization protocol was specifically designed to empower nurses to autonomously manage ACT measurements and repeat heparin administration without the need for interruptions of the operating physician for heparin dosing decisions. The protocol was developed as an institutional initiative based on current guideline recommendations for intraprocedural anticoagulation (target ACT > 300 s), combined with internal clinical experience and previously published weight‐based and ACT‐guided dosing strategies. The protocol follows a three‐phase approach. In Phase 1, an initial fixed bolus of 5000 IU of UFH was administered immediately after femoral venous access but before transseptal puncture. Phase 2 involved a weight‐based supplemental bolus of 1000 IU for every 10 kg of body weight above 50 kg, given directly after transseptal puncture. In Phase 3, ACT was measured autonomously by nurses every 20 min. According to measured ACT and bodyweight, fixed boli of heparin were administered by nurses as specified by the heparinization protocol.

### 2.3 Nurse Training

Prior to institutional implementation of the heparin protocol, all electrophysiology nurses underwent a structured training program covering the basic pharmacology of UFH, principles of ACT monitoring, and details of the dosing algorithm. The program included didactic sessions and supervised application of the protocol in live cases. Competency was evaluated using a formal checklist assessing accurate dose calculation, correct ACT interpretation, and adherence to the algorithm. Nurses were certified for independent protocol application after successfully managing at least 10 supervised cases without error. All anticoagulation management within the protocol was performed by the electrophysiology‐trained nursing staff only. No anesthesia personnel were involved in protocol execution.

### 2.4 ACT Measurement Technology

All ACT values were obtained using a point‐of‐care testing device (Hemochron Signature Elite, Accriva Diagnostics). ACT samples were drawn from the sheath in the femoral venous access site using a standard technique, ensuring avoidance of heparin contamination. Quality control checks were performed daily on the Hemochron system, and any out‐of‐range quality control results prompted device recalibration before clinical use.

### 2.5 Periprocedural Management

All ablation procedures were conducted under deep sedation using intravenous propofol, midazolam, and fentanyl. Preprocedurally, all patients underwent contrast‐enhanced computed tomography (CT) of the left atrium and pulmonary veins to delineate individual pulmonary vein anatomy. For anticoagulation management, patients on direct oral anticoagulants (DOACs) withheld the morning dose on the day of the procedure and resumed therapy in the evening following ablation. In those patients receiving vitamin K antagonists prior to ablation, treatment was continued uninterrupted maintaining an international normalized ratio (INR) between 2.0 and 3.0 without bridging medication. During the procedure, UFH was administered in all patients targeting ACT levels of ≥ 300 s. Following ablation, all venous sheaths were removed, and hemostasis was achieved using a figure‐of‐eight suture at each femoral access site. A transthoracic echocardiogram (TTE) was performed in all patients prior to leaving the electrophysiology laboratory to exclude pericardial effusion. Patients were subsequently transferred to a monitored recovery area. Hospital discharge was routinely planned for the following day, after removal of groin sutures and repeat TTE confirming the absence of pericardial effusion.

### 2.6 Ablation Procedures

All ablation procedures were performed using one of three energy sources: radiofrequency ablation (RFA), cryoballoon ablation (CBA), or pulsed field ablation (PFA). Cryoballoon and pulsed field ablation were preferentially selected for patients undergoing their first ablation procedure for AF. RF ablation was mainly performed for repeat AF ablation or for patients who presented with organized AT. Balloon‐based cryoablation and PFA were performed using commercially available systems (POLARx, Boston Scientific, St Paul, MN, USA; Arctic Front Advance Pro, Medtronic Inc., Minneapolis, MN, USA; Farapulse, Boston Scientific, Menlo Park, CA, USA). Femoral venous access was obtained according to institutional standard practice, with ultrasound guidance used in all patients. This approach was applied consistently throughout the study period and did not differ between the study and control groups. For balloon‐based cryoablation and PFA femoral, groin access was established using one eight‐French and one seven‐French sheath. Patients treated with balloon‐based cryoablation received a single transseptal puncture (SL1, St. Jude Medical Inc., St. Paul, MN, USA). After transseptal puncture, the SL1 sheath was exchanged for the device sheath (POLARSHEATH, Boston Scientific, St Paul, MN, USA; FlexCath Advance, Medtronic Inc., Minneapolis, MN, USA). The detailed institutional ablation approach has been published before [11]. In patients undergoing PFA, a single transseptal puncture was performed using a SL1 sheath or the dedicated steerable device sheath (FARADRIVE, Boston Scientific, Menlo Park, CA, USA). All PFA procedures were guided by three‐dimensional electroanatomic mapping systems (EnSite X, Abbott, Abbott Park, IL, USA; OPAL HDx, Boston Scientific, St Paul, MN, USA) with a dedicated mapping catheter (Advisor FL, Abbott, Abbott Park, IL, USA; Orion TM, Boston Scientific, St Paul, MN, USA) used for pre‐ and post‐ablation atrial voltage mapping. Detailed ablation parameters have been previously described [12, 13]. In patients undergoing RF ablation procedures, femoral groin access was established using two right‐sided eight‐French and two left‐sided seven‐French sheaths. Operators performed either a single or double transseptal puncture based on anatomic considerations and procedural strategy. Three‐dimensional electroanatomic mapping was performed with either a spiral catheter (Advisor FL, Abbott, Abbott Park, IL, USA) or a high‐density mapping catheter (HD Grid, Abbott, Abbott Park, IL, USA). Ablation was delivered using a sensor‐enabled irrigated ablation catheter (TactiFlex SE, Abbott, Abbott Park, IL, USA) with a power setting of 50 W. The procedural endpoint of RFA, CBA, or PFA procedures was confirmation of entrance and exit block in all pulmonary veins. Additional left atrial substrate modification in patients undergoing RF‐based AF ablation was at the operator's discretion and performed as linear lesion ablation according to institutional standards. In cases where left atrial linear lesion ablation was conducted, differential pacing maneuvers were performed to confirm bidirectional block across ablation lines as described before [14].

### 2.7 Study Group: Standardized Heparin Dosage Protocol

**Figure 2:**
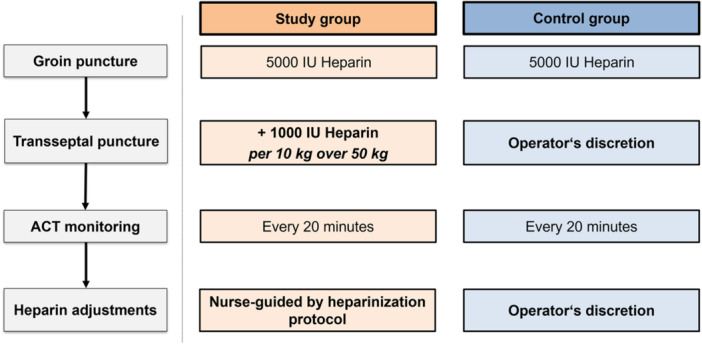
Study protocol for heparin administration and monitoring. Study protocol for periprocedural anticoagulation in the study group versus the control group. In both groups, an initial bolus of 5000 IU heparin was administered after groin puncture. In the study group, an additional 1000 IU heparin was given per 10 kg body weight above 50 kg, followed by ACT monitoring every 20 min and nurse‐guided adjustments according to the standardized heparin scheme. In the control group, supplemental heparin dosing and adjustments were at the operator's discretion, with ACT monitoring every 20 min.

In the study group, intraprocedural anticoagulation management after transseptal puncture was autonomously performed by the assisting nurses (Figures 1 and 2). An initial bolus of 5000 IU of heparin was administered after femoral venous access by the operator. After transseptal puncture, a supplemental bolus of 1000 IU heparin was administered for every 10 kg of body weight above 50 kg. From then on, ACT was measured at 20 min intervals by the assisting nurses. In patients with ACT levels < 300 s, the assisting nurses repeatedly administered the heparin dose as specified in the heparinization protocol without further consultation of the operator.

### 2.8 Control Group: Operator‐Guided Anticoagulation

In the control group, intraprocedural anticoagulation management was at the discretion of the operating physician (Figure 2). Typically, this involved an initial bolus of 5000 IU of heparin administered after femoral venous access, followed by additional boluses as deemed necessary by the operator, without adherence to a formal dosing algorithm. ACT was monitored at 20 min intervals.

### 2.9 Data Collection and Endpoints

For all patients, demographic and clinical characteristics were recorded prospectively, together with detailed procedural information, including ablation modality, total procedural duration, and fluoroscopy time. Anticoagulation parameters were also collected, encompassing the timing and dosage of heparin boluses, all ACT measurements, and the interval from heparin administration to achievement of an ACT greater than 300 s. The primary endpoint was the proportion of patients in whom at least one intraprocedural ACT measurement exceeded 300 s at any time during the procedure. Secondary endpoints comprised the time from the first heparin bolus to the attainment of an ACT above 300 s, the variability of ACT values—expressed as the standard deviation of ACT measurements within each case—the total number of physician‐administered heparin boluses following transseptal puncture, and the occurrence of procedural safety events. Safety outcomes included major bleeding, defined as bleeding that required transfusion or intervention; minor bleeding, defined as access‐site hematomas not requiring intervention; thromboembolic complications, including stroke, TIA, or systemic embolism; and pericardial effusion or tamponade requiring drainage.

### 2.10 Statistical Analysis

All data were analyzed using SPSS version 28 (IBM Corp., Armonk, NY). Continuous variables were assessed for normality using the Shapiro–Wilk test and are presented as mean ± standard deviation. Group comparisons were made using Student's *t*‐test for normally distributed variables or the Mann–Whitney *U* test for non‐normally distributed variables. Categorical variables were expressed as frequencies and percentages and compared using the chi‐square test or Fisher's exact test as appropriate. A two‐sided *p* < 0.05 was considered statistically significant.

## 3 Results

### 3.1 Baseline Characteristics

A total of 1310 patients were included, with 655 assigned to the study group and 655 to the control group. Baseline characteristics were similar between groups (Table 1). The mean age was 65.2 ± 11.0 years in the study group and 64.9 ± 11.5 years in the control group (*p *= 0.62); 33.2% and 33.7% of patients were female, respectively (*p *= 0.98). The mean body‐mass index (BMI) was 28.7 ± 5.5 kg/m² in the study group and 28.8 ± 6.0 kg/m² in the control group (*p *= 0.75). Median CHA₂DS₂‐VASc scores were 2 (IQR 1–3) and 3 (IQR 1–4) (*p *= 0.54), and median HAS‐BLED scores were 1 (IQR 1–2) and 2 (IQR 1–2) (*p *= 0.13) in the study and control groups, respectively. A history of major bleeding was reported in 0.2% and 0.8% (*p *= 0.10), and a history of stroke or TIA in 9.0% and 10.1% (*p *= 0.51). A history of coagulation disorder was present in 1.2% and 1.5% of patients (*p *= 0.64). Oral anticoagulation was used by 86.0% of study‐group patients and 83.6% of control‐group patients (*p *= 0.58); DOACs were most frequent (80.3% vs. 78.6%, *p* = 0.45). Reduced‐dosage DOACs were prescribed in 2.1% and 2.3% (*p *= 0.85). Antiplatelet therapy was used by 2.4% and 2.3% (*p *= 0.86). Paroxysmal AF was present in 33.9% and 32.4% (*p *= 0.35), and persistent AF in 61.8% and 57.7% (*p *= 0.13). Baseline characteristics are summarized in Table 1.

**Table 1:** Baseline data.

	Study group	Control group	*p*
Patients, *n*	655	655	
Age [years]	65.2 ± 11.0	64.9 ± 11.5	0.62
Female, *n* (%)	218 (33.2)	221 (33.7)	0.98
BMI [kg/m²]	28.7 ± 5.5	28.8 ± 6.0	0.75
CHA_2_DS_2_‐VASC score	2 {1; 3}	3 {1; 4}	0.54
HAS‐BLED score	1 {1; 2}	2 {1; 2}	0.13
History of major bleeding, *n* (%)	1 (0.2)	5 (0.8)	0.10
History of stroke/TIA, *n* (%)	59 (9.0)	66 (10.1)	0.51
Coagulation disorder, *n* (%)	8 (1.2)	10 (1.5)	0.64
Thrombocytopenic disorder	1 (0.15)	3 (0.5)	0.32
Thrombophilic disorder	7 (1.1)	7 (1.1)	1.0
Oral anticoagulation, *n* (%)	563 (86.0)	556 (83.6)	0.58
DOAC	526 (80.3)	515 (78.6)	0.45
Vitamin K antagonists	37 (5.6)	41 (6.3)	0.64
Reduced dosage of DOAC	14 (2.1)	15 (2.3)	0.85
Antiplatelet therapy, *n* (%)	16 (2.4)	15 (2.3)	0.86
Type of arrhythmia, *n* (%)			
Paroxysmal AF	222 (33.9)	212 (32.4)	0.35
Persistent AF	405 (61.8)	378 (57.7)	0.13
Atrial tachycardia, *n* (%)	25 (3.8)	30 (4.6)	0.58

Abbreviations: AF, atrial fibrillation; BMI, body mass index; DOAC, direct oral anticoagulant; TIA, transient ischemic attack.

### 3.2 Procedural Characteristics

Procedural parameters are shown in Table 2. Pulmonary vein isolation (PVI) alone was performed in 76.6% of study‐group procedures and 73.4% of control‐group procedures (*p* = 0.20); PVI combined with additional left atrial substrate modification was performed in 23.4% and 22.0% (*p* = 0.59), respectively. Among ablation modalities, RF ablation was performed in 52.7% and 52.2% (*p* = 0.12), cryoballoon PVI in 25.9% and 22.3% (*p* = 0.65), and pulsed‐field ablation in 21.5% and 25.5% (*p* = 0.01) of study and control procedures. The mean total amount of heparin administered was 12570.7 ± 4905.0 and 11730.3 ± 3238.7 IU (*p* = 0.0003). The mean number of ACT measurements per procedure was 2.2 ± 1.2 in the study group and 2.3 ± 1.1 in the control group (*p* = 0.12).

**Table 2:** Procedural data.

	Study group	Control group	*p*
Type of procedure, *n* (%)			
PVI only	502 (76.6)	481 (73.4)	0.20
PVI + substrate modification/LAT	153 (23.4)	144 (22.0)	0.59
Type of ablation, *n* (%)			
Radiofrequency PVI/ablation	313 (52.7)	342 (52.2)	0.12
Cryoballoon PVI	154 (25.9)	146 (22.3)	0.65
Pulsed field ablation	128 (21.5)	167 (25.5)	0.01
Total number of ACT tests	2.2 ± 1.2	2.3 ± 1.1	0.12
Total amount of heparin [IU]	12 570.7 ± 4905.0	11 730.3 ± 3238.7	0.0003

Abbreviations: ACT, activated clotting time; IU, international units; LAT, linear ablation therapy; PVI, pulmonary vein isolation.

### 3.3 ACT Findings

**Figure 3:**
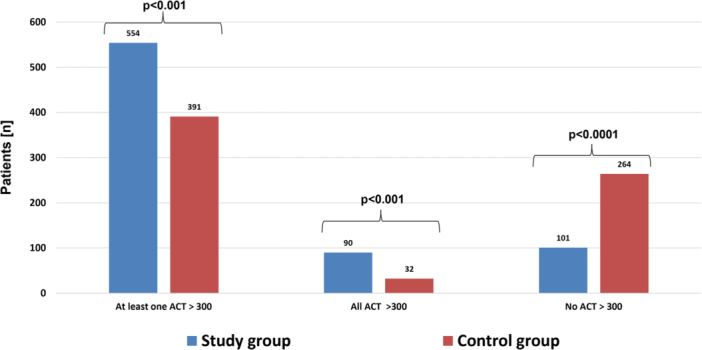
Anticoagulation effectiveness in study and control groups. Comparison of ACT > 300 s achievement between the study group and control group. The study group showed a higher proportion of patients with at least one ACT > 300 s and all ACT values > 300 s, and fewer patients with no ACT > 300 s, compared to the control group (*p* < 0.001 for all comparisons).

**Figure 4:**
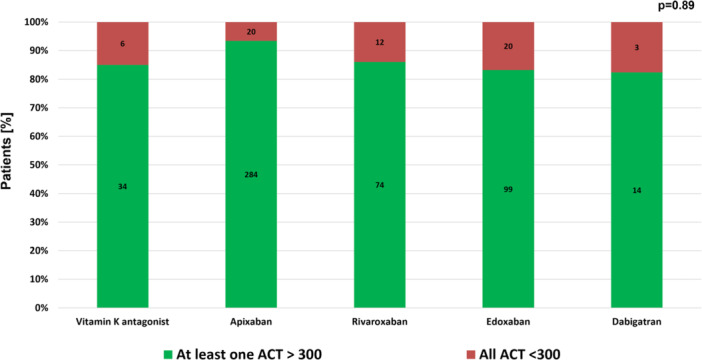
Anticoagulation effectiveness in the study group according to baseline anticoagulant. Achievement of ACT ≥ 300 s stratified by preprocedural anticoagulant type. The proportion of patients with at least one ACT ≥ 300 s (green) versus all ACT values < 300 s (red) is shown for vitamin K antagonists, apixaban, rivaroxaban, edoxaban, and dabigatran. There was no statistically significant difference in achievement of ACT levels ≥ 300 s between patients receiving direct oral anticoagulation with apixaban, rivaroxaban, edoxaban, or dabigatran compared to patients receiving vitamin k antagonists (*p* = 0.89).

Overall ACT results are summarized in Figure 3. At least one ACT measurement greater than 300 s was observed in 554 patients (84.6%) in the study group and in 391 (59.7%) in the control group (*p *< 0.001). There was no significant difference between different anticoagulation regimes (vitamin K antagonists and DOACs, *p* = 0.89) with regard to achievement of at least one therapeutic ACT level (Figure 4). All ACT values were above 300 s in 90 (13.7%) and 32 (4.9%) patients (*p* < 0.001), respectively. No ACT measurement greater than 300 s occurred in 101 (15.4%) study‐group patients and in 264 (40.3%) control‐group patients (*p* < 0.0001). ACT values exceeding 400 s occurred in 13 of 655 patients (2.0%) in the study group compared with 33 of 655 patients (5.0%) in the control group (*p* = 0.0027).

Among patients undergoing RFA, 93% of ACT measurements exceeded 300 s in the study group, as compared with 77% in the control group (*p* < 0.001). In cryoballoon procedures, ACT values above 300 s were observed in 90% of patients in the nurse‐led group and in 75% of those in the operator‐guided group (*p* = 0.01). Among patients undergoing PFA, ACT values above 300 s were observed in 91% of patients in the study group and 69% in the control group (*p* = 0.0002). The mean time to achieve an ACT greater than 300 s was 26 ± 11.1 min in the nurse‐led group and 42 ± 10.8 min in the operator‐guided group (*p* < 0.001) among patients receiving vitamin K antagonists. For patients treated with DOACs, the corresponding times were 25 ± 11 min and 36 ± 13 min, respectively (*p* < 0.001). In the subgroup of patients with a BMI ≥ 30 kg/m², therapeutic ACT was reached after 30 ± 12.9 min in the nurse‐led group and 41 ± 14 min in the control group (*p* < 0.001).

### 3.4 Complications

**Figure 5:**
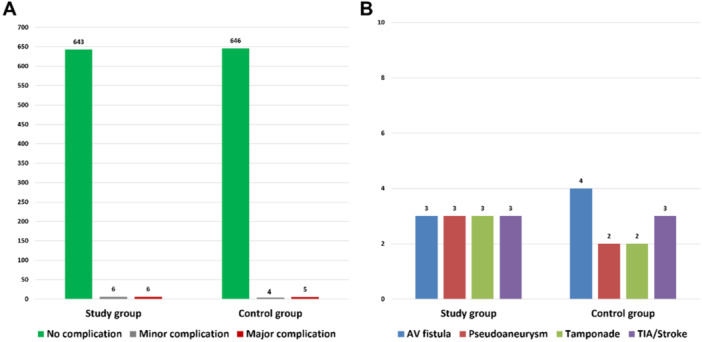
Safety outcomes. Periprocedural complications in the study group versus the control group. (A) Distribution of no complication (green), minor complication (gray), and major complication (red) cases in both groups. (B) Specific complication types: arteriovenous (AV) fistula, pseudoaneurysm, tamponade, and transient ischemic attack or stroke. The frequency of each complication type is shown for the study and control groups. No statistically significant difference was observed between the study and control groups for the occurrence of complications in general or specific complications.

Periprocedural complications are summarized in Figure 5A,B. Complications occurred in 12 of 655 patients (1.8%) in the study group and in 9 of 655 patients (1.4%) in the control group, with no significant difference between groups (*p *= 0.66). In the study group, 643 patients (98.2%) had no complications, 6 (0.9%) patients experienced minor complications, and 6 patients (0.9%) had major complications. In the control group, 646 patients (98.6%) had no complications, 4 patients (0.6%) experienced minor complications, and 5 patients (0.8%) had major complications. The distribution of specific complications is shown in Figure 5B. Arteriovenous fistula occurred in 3 patients (0.5%) in the study group and 4 patients (0.6%) in the control group. Pseudoaneurysm was observed in 3 patients (0.5%) and 2 patients (0.3%), respectively. Pericardial tamponade occurred in 3 patients (0.5%) in the study group and 2 patients (0.3%) in the control group. TIA or stroke was documented in 3 patients (0.5%) in each group. All patients with TIA or stroke had at least one ACT > 300 s during the ablation procedure. No cases of heparin‐induced thrombocytopenia were identified during hospitalization or early follow‐up.

## 4 Discussion

This study yielded several key findings regarding the use of a standardized, nurse‐led heparinization protocol during left atrial ablation. The principal results are the following:

1. Therapeutic intraprocedural anticoagulation was achieved more frequently. A significantly greater proportion of patients in the study group reached an ACT exceeding 300 s, as compared with those managed at the operator's discretion.
2. Target ACT levels were achieved faster. Time to therapeutic anticoagulation was consistently shorter across all baseline anticoagulant regimens and across all patient subgroups.
3. The stability of anticoagulation was higher. Patients in the study group were less likely to have exclusively subtherapeutic ACT values, and intraprocedural ACT variability was reduced.
4. The incidence of excessive anticoagulation was lower. ACT values above 400 s occurred markedly less often in the study group, indicating tighter control of heparin dosing.
5. Procedural complications did not increase. Despite more consistent and precise anticoagulation, rates of bleeding, vascular complications, pericardial tamponade, and thromboembolic events were similar in the two groups.

Taken together, these findings indicate that a structured, algorithm‐driven approach to intraprocedural anticoagulation—implemented and executed by trained nursing staff—enhances both the efficacy and precision of heparin administration without compromising safety. The protocol improved attainment and maintenance of therapeutic ACT levels while minimizing supratherapeutic excursions, thereby providing more reliable anticoagulation during left atrial ablation.

### 4.1 Comparison With Prior Studies and Guidelines

Our results align with growing evidence that standardized heparinization protocols improve intraprocedural anticoagulation during left atrial ablation. A recent study by Ural et al. demonstrated that an anesthesia‐driven, algorithm‐based UFH protocol significantly increased time in the therapeutic ACT range compared to non‐standardized operator‐directed dosing (84.9% vs. 75.8%, *p* < 0.001), while also improving first‐draw therapeutic ACT achievement (70.3% vs. 31.2%, *p* < 0.001) without increasing bleeding or thromboembolic events requiring intervention [15]. These findings mirror the anticoagulation consistency and safety profile observed in our study, supporting the concept that structured dosing algorithms enhance control early after transseptal access, a recognized period of thromboembolic vulnerability [3, 5, 10, 16, 17]. Importantly, Ural et al. extended protocol autonomy to the anesthesia team, adjusting bolus dosing according to baseline ACT and scaling dosing weight in patients with BMI > 35 kg/m², highlighting the physiologic limitations of both ideal‐weight‐based and uncapped actual‐weight‐based heparin dosing [6, 15, 16, 17]. Similar to our experience, the protocol was developed in response to institutional frustration with provider‐to‐provider variability and demonstrates a philosophical and practical shift toward multidisciplinary anticoagulation stewardship [6, 7, 17].

To our knowledge, this is one of the most recent cohorts showing that a standardized UFH protocol improves ACT therapeutic performance without compromising clinical safety, reinforcing current guideline emphasis on procedural ACT targets (> 300–350 s), while also demonstrating that protocol‐driven UFH dosing can be successfully managed by non‐operator teams without increased complications.

Our findings are consistent with current guideline recommendations, which emphasize the importance of maintaining ACT values above 300 s to mitigate thromboembolic risk during left atrial ablation [1, 2]. Previous observational studies have linked subtherapeutic ACT to increased rates of silent cerebral embolic lesions and clinically apparent thromboembolism [10]. Despite these recommendations, intraprocedural heparin dosing in real‐world practice remains highly variable, often relying on physician discretion and resulting in inconsistent ACT trajectories—particularly during the vulnerable early period following transseptal puncture [5, 8, 16, 17]. To our knowledge, this is the first study to systematically evaluate a fully nurse‐led anticoagulation protocol in electrophysiology. Successful nurse‐driven protocols have been reported in other procedural settings, including interventional cardiology and intensive care, where structured algorithms have improved therapeutic consistency and reduced dosing errors [6, 7]. The present study extends this paradigm to left atrial ablation and demonstrates that carefully trained nursing staff can deliver anticoagulation management that is at least equivalent—and in several respects superior—to traditional operator‐driven approaches. Recent evidence has also demonstrated that ACT behavior differs between PFA and traditional thermal modalities, underscoring the importance of standardized anticoagulation approaches across energy sources [18].

### 4.2 Considerations Regarding Body Weight and Heparin Response

Beyond the overall improvement in anticoagulation performance, our data highlight an important interaction between body weight and time to therapeutic ACT. Heparin pharmacokinetics show substantial interpatient variability driven in part by BMI and body composition [6]. In the study group, therapeutic ACT was reached more rapidly across all weight categories, including patients with a BMI ≥ 30 kg/m², a group traditionally considered more challenging for heparin dosing [9]. Nevertheless, individuals with a BMI > 35 kg/m² experienced slightly delayed achievement of therapeutic ACT compared with those with lower body weight, suggesting that the current weight‐adjusted bolus strategy may not fully account for heparin requirements in patients with extreme obesity [6, 9]. Given that delays in achieving therapeutic ACT early after transseptal puncture may increase thromboembolic vulnerability, these observations have practical implications. While the standardized protocol clearly improves performance across a broad patient population, future refinements—potentially involving steeper weight‐based increments or early ACT‐guided bolus adjustments—may further optimize anticoagulation in patients with very high body weight. A notable secondary finding was the significantly lower occurrence of ACT values exceeding 400 s in the protocol group. Avoiding such supratherapeutic excursions is clinically relevant, as excessive anticoagulation may increase the risk of bleeding and pericardial complications [3, 19]. The reduction in ACT > 400 s indicates that the protocol not only accelerates achievement of therapeutic levels but also enhances dosing precision by preventing unnecessary heparin administration [6, 7]. Importantly, these benefits did not come at the expense of safety.

### 4.3 Safety Interpretation

Despite more consistent anticoagulation and fewer extremes of overanticoagulation, overall procedural safety remained unchanged. Rates of major bleeding, vascular complications, pericardial tamponade, and thromboembolic events were low in both groups and did not differ significantly. This neutral safety signal suggests that tighter anticoagulation control does not predispose patients to hemorrhagic or mechanical complications. Multiple studies have shown that rapid achievement of ACT > 300 s does not increase major bleeding within the ACT ranges typically used for ablation [4, 19]. Nevertheless, because the absolute number of events was small, the study was not powered to detect differences in rare outcomes such as stroke or tamponade [3]. Larger multicenter evaluations will be necessary to determine whether reducing ACT variability can translate into measurable improvements in clinical safety endpoints.

### 4.4 Implications for Workflow and Cognitive Load

A key advantage of the nurse‐led protocol lies in its impact on workflow and cognitive burden. Left atrial ablation requires sustained operator concentration for mapping and lesion delivery. Conventional anticoagulation management interrupts this process, obliging the operator to periodically re‐engage with ACT interpretation and heparin dosing decisions. Prior work has described how anticoagulation management imposes workflow and attention burdens on operators [6, 17]. By delegating these tasks to trained nurses following a standardized algorithm, physicians can remain focused on critical procedural components, a shift that may reduce distractions, procedural errors, and cognitive load. This expansion of nursing responsibilities represents a meaningful evolution in electrophysiology laboratory practice. The protocol fosters greater autonomy within the nursing team, supported by structured training and competency verification. Such models align with broader trends in healthcare that emphasize interprofessional collaboration and expanded nursing scope of practice [6, 7, 20]. Furthermore, empowerment of nursing staff may enhance workforce resilience—an increasingly relevant goal in the context of global staffing shortages.

Although this study did not include a formal cost analysis, the workflow efficiencies observed suggest potential economic benefits. Reduced interruptions may shorten procedures, increase case throughput, and improve utilization of high‐cost laboratory resources. Minimizing ACT variability may also reduce downstream costs associated with complications. Future studies incorporating time‐motion assessments and cost‐effectiveness analyses will be valuable.

### 4.5 Generalizability

The generalizability of these findings warrants consideration. This protocol was introduced in a high‐volume electrophysiology center with experienced nursing staff and well‐established procedural workflows, elements that likely facilitated its successful implementation. Nevertheless, the dosing algorithm is straightforward, relies only on weight‐based calculations and ACT thresholds, and does not require specialized equipment. With appropriate training and institutional support, the protocol is potentially scalable to a wide range of centers, including those with varying volumes and staffing structures. Prospective implementation studies in diverse environments will be important to validate broader applicability.

### 4.6 Clinical Implications

From a clinical standpoint, these results support the adoption of structured anticoagulation algorithms as part of routine practice in electrophysiology laboratories. A nurse‐led, protocol‐driven approach offers a reproducible method to achieve more consistent and reliable intraprocedural anticoagulation while reducing operator workload, consistent with evidence that protocol‐driven anticoagulation improves ACT control compared with ad hoc management [6, 7, 17]. Institutions seeking to enhance procedural standardization, improve workflow, and strengthen multidisciplinary engagement may find this model particularly advantageous. Broader implementation of such protocols has the potential to elevate procedural quality and safety across a variety of ablation settings.

### 4.7 Limitations

This study has several limitations. First, it is an observational single‐center study, which may limit generalizability and introduce the possibility of residual confounding. Second, the comparison of two consecutive time periods introduces a potential temporal bias, as procedural experience and technologies may have evolved over time. Third, different ablation energy sources were used, including radiofrequency, cryoballoon, and pulsed field ablation, which may influence procedural duration and anticoagulation dynamics. Finally, although complication rates were low and comparable between groups, the study was not powered to detect differences in rare safety endpoints such as stroke or pericardial tamponade.

## 5 Conclusions

Implementation of a standardized, nurse‐led heparinization protocol during left atrial ablation improved the frequency, timing, and stability of achieving therapeutic ACT levels while reducing excessive anticoagulation. These benefits were obtained without an increase in procedural complications. A structured, algorithm‐based approach allows trained nursing staff to manage intraprocedural anticoagulation safely and effectively, supporting broader adoption of nurse‐driven protocols to enhance procedural consistency and workflow in electrophysiology laboratories.

## Conflicts of Interest

The authors declare no conflicts of interest.

## Data Availability

The data that support the findings of this study are available from the corresponding author upon reasonable request.
